# Diabetic Ketoacidosis, Very Severe Hypertriglyceridemia, and Pseudohyponatremia Successfully Managed With Insulin Infusion

**DOI:** 10.7759/cureus.9306

**Published:** 2020-07-20

**Authors:** Ramzi Ibrahim, Mohammed Salih, Chirine Elmokdad, Amreetpal Sidhu

**Affiliations:** 1 Internal Medicine, St. Joseph Mercy Oakland Hospital, Pontiac, USA

**Keywords:** triglyceride, insulin, pancreatitis

## Abstract

Hypertriglyceridemia is a common lipid abnormality that has serious consequences, such as acute pancreatitis and premature atherosclerosis. The consensus for first-line treatment to lower the triglyceride levels has not been fully evaluated. We present a case of very severe hypertriglyceridemia with diabetic ketoacidosis and an artifactual pseudohyponatremia. The patient was effectively and safely treated with guideline-directed medical therapy; however, it needed a longer duration of intravenous insulin. Therefore, our case has been in agreement with literature by concluding that insulin is in fact an effective and minimally invasive form to lower a high triglyceride level, especially in patients who have concurrent uncontrolled diabetes mellitus.

## Introduction

Hypertriglyceridemia is one of the most common lipid abnormalities. It can be primary (genetic, type I, IV, or V) or secondary to other causes (obesity, drug side-effects, pregnancy, alcoholism, and diabetes mellitus). In the US, it is estimated >4 million individuals have triglyceride levels greater than 500 mg/dL [1,2]. Severe hypertriglyceridemia is defined as a triglyceride level of at least 1,000 mg/dL, occurring in <1 in 5,000 individuals [3]. However, very severe hypertriglyceridemia, defined as levels above 2,000 mg/dL, is estimated to be about 1.8 per 10,000 white adults [4]. Once triglyceride levels reach above 1,500 mg/dL, the risk of pseudohyponatremia significantly rises. This represents an artifactual reduction of blood sodium levels due to the inaccuracy of indirect potentiometry. Increases in various components of the bloodstream including lipids, glucose, and proteins may cause this finding. The first-line treatment of hypertriglyceridemia to mitigate complications such as acute pancreatitis has not been established in literature; however, insulin shows a beneficial rapid effect. Below, we present a case of diabetic ketoacidosis and very severe hypertriglyceridemia with artifactual pseudohyponatremia which was effectively managed with insulin therapy.

## Case presentation

A 45-year-old African American female with a pertinent history of hypertension presented to the hospital with a severe headache of two-day duration. It was a bandlike headache, throbbing in nature, and 8 out of 10 on a pain severity scale. Tylenol helped minimally with the pain and physical activity made it worse. The patient denied fever, chills, cough, rhinorrhea, tearing, aura, phonophobia, photophobia, visual symptoms, nausea, or vomiting.

Physical examination revealed vitals significant for a heart rate of 101 and regular, respiratory rate of 24 breath/min, blood pressure of 153/98 mmHg, and temperature at 98.1˚F. The general exam showed an overweight female with acidotic breathing with an otherwise normal physical exam. The laboratory results were significant for a sodium level of 113 mEq/L, chloride level of 87 mEq/L, bicarbonate level of 13 mEq/L, and an anion gap of 15 mEq/L. Serum osmolarity was within normal limits. Urinalysis was positive for glucose and ketones. Complete blood count (CBC) and lipase levels were unremarkable. Total cholesterol was measured at 210 mg/dL, high-density lipoprotein (HDL) at 12 mg/dL, and triglyceride level >5,000 mg/dL.

The patient was admitted and treated for diabetic ketoacidosis and hypertriglyceridemia with intravenous (IV) insulin and IV fluid. The patient was also started on high-intensity atorvastatin at 80 mg/day and fenofibrate 54 mg/day. The sodium level was corrected with a goal of 6-8 mmol/L/day. She was kept nothing by mouth (NPO) initially with an eventual transition to a low-fat diet to prevent ketosis. The patient was monitored closely in the intensive care unit for signs and symptoms of pancreatitis.

On the second day, glucose was within normal limits and the anion gap closed. The patient was kept on IV insulin to lower the triglyceride levels. Subsequently, insulin was bridged to subcutaneous insulin on the fifth day of hospitalization. On the sixth day of hospitalization, triglyceride levels finally decreased to 707 mg/dL. The sodium level was noted to be 133 mEq/L while the glucose was within normal limits. On the seventh day, the patient was discharged on statin and fibrate therapy, educated on diabetic control as this was newly diagnosed, and scheduled to follow up in two weeks.

## Discussion

Hypertriglyceridemia can be asymptomatic or symptomatic with potentially life-threatening complications. Signs and symptoms include eruptive xanthomas, xanthelasma, lipemia retinalis, hepatosplenomegaly, and abdominal pain signifying pancreatitis. Toxic-free fatty acids, from the breakdown of triglycerides by pancreatic lipase, may cause lipotoxicity. This contributes to pancreatic damage and impeding acute pancreatitis. This finding is prominent in those with triglycerides over 2,000 mg/dL, estimated in about 10%-20% of cases [5]. Another finding associated with severe hypertriglyceridemia may include pseudohyponatremia, as seen in our patient.

Managing severe/very severe hypertriglyceridemia is paramount to avoid unnecessary complications. This typically requires both dietary modifications and the use of medications that aid in the decrease of triglyceride levels. During the acute phase of management to decrease triglyceride levels, patients should be kept NPO with the eventual advancement of the diet.

Lipoprotein lipase (LPL) is an enzyme produced by capillary endothelial cells in adipose tissue and muscle. The function of this enzyme is to hydrolyze triglycerides into two basic components; glycerol and fatty acids. This enzyme’s activity can be augmented with the use of IV insulin and has been hypothesized to be effective when attempting to rapidly lower triglyceride levels. The insulin can be given with or without glucose depending if diabetes mellitus is a comorbid condition. In our patient, we effectively decreased the triglyceride levels by giving insulin over a seven-day period. There have been no studies that indicate the preferred route of administration of insulin (IV versus subcutaneous) for the treatment of hypertriglyceridemia. This practice of giving insulin was supported by various studies, including a case series report by Coskun et al. indicating that insulin therapy was indeed beneficial in treating hypertriglyceridemia even in the presence of acute pancreatitis [6]. However, there is an ongoing debate regarding the effectiveness of using plasmapheresis instead of insulin. Plasmapheresis has a greater direct cost compared to insulin, estimated about a 100-fold cost difference between the two methods [7]. Also, plasmapheresis is labor intensive and has a greater myriad of potential complications associated with it, whereas insulin is minimally invasive and is beneficial in cases where diabetes mellitus also exists. Similarly, a prospective, randomized controlled trial in 2016 showed that high-volume hemofiltration resulted in lower triglyceride levels when compared to insulin, but there was no improvement in clinical outcome [8]. The Bi-TPAI trial, which is a multicenter, parallel-group, randomized controlled trial of 220 patients, scheduled to be completed by November 2020, has assigned some patients with hypertriglyceridemia-induced acute pancreatitis to the IV insulin arm and some to the plasmapheresis arm [9]. This study’s primary endpoint is a triglyceride level below 500 mg/dL. The results of this study would help steer medical literature in the right direction for the agreement of the first-line treatment of hypertriglyceridemia.

Other medications that can be used for the treatment of hypertriglyceridemia include fibrates. These drugs have a slower onset of action and may take a few weeks to show its maximal benefit. It is not considered a treatment for a rapid lowering of triglyceride levels. Also, if concurring hypercholesterolemia exists, statins may also be initiated. However, a combination of fibrates and statin medications increases the risk of myopathy and should, therefore, be assessed.

The goal of reduction in triglyceride levels should be below 1,000 mg/dL which effectively decreases the risk of acute pancreatitis. Patients should then be maintained on a no-fat or low-fat diet, which decreases the risk of postprandial chylomicronemia due to already saturated LPL from triglyceridemia.

## Conclusions

The risk of acute pancreatitis in patients with a triglyceride level above 1,500 mg/dL is significantly high, but not 100%. Our patient had no stigmata of acute pancreatitis. The general goal of therapy for patients with a remarkably high triglyceride level would be to rapidly decrease the level to avoid complications. This includes diet, pharmacological therapy, including insulin, and avoiding medications that may induce rises in the triglyceride levels such as oral estrogens, antiretroviral regiments, second-generation antipsychotics, diuretics, glucocorticoids, and more. Our findings have been in agreement with the literature. Treatment with insulin is a minimally invasive form and effective therapy for hypertriglyceridemia.
